# Structures of the NLRP14 pyrin domain reveal a conformational switch mechanism regulating its molecular interactions

**DOI:** 10.1107/S1399004714010311

**Published:** 2014-06-29

**Authors:** Clarissa Eibl, Manuel Hessenberger, Julia Wenger, Hans Brandstetter

**Affiliations:** a Department of Molecular Biology, University of Salzburg, Billrothstrasse 11, 5020 Salzburg, Austria

**Keywords:** pyrin domain, NLRP14

## Abstract

The cytosolic tripartite NLR receptors serve as important signalling platforms in innate immunity. While the C-terminal domains act as sensor and activation modules, the N-terminal death-like domain, *e.g.* the CARD or pyrin domain, is thought to recruit downstream effector molecules by homotypic interactions. Such homotypic complexes have been determined for all members of the death-domain superfamily except for pyrin domains. Here, crystal structures of human NLRP14 pyrin-domain variants are reported. The wild-type protein as well as the clinical D86V mutant reveal an unexpected rearrangement of the C-terminal helix α6, resulting in an extended α5/6 stem-helix. This reordering mediates a novel symmetric pyrin-domain dimerization mode. The conformational switching is controlled by a charge-relay system with a drastic impact on protein stability. How the identified charge relay allows classification of NLRP receptors with respect to distinct recruitment mechanisms is discussed.

## Abstract 2

Pyrin domains (PYDs) recruit downstream effector molecules in NLR signalling. A specific charge-relay system suggests a the formation of a signalling complex involving a PYD dimer.

## 1. Introduction   

The cytosolic nucleotide-binding domain leucine-rich repeat-containing receptors (NLRs) play prominent roles in innate immunity (Kufer *et al.*, 2005 ▶; Kersse, Bertrand *et al.*, 2011 ▶). In the last decade, NLRs have been identified as germ-line-encoded multi-domain pattern-recognition receptors and have been greatly studied since then (for a review, see Kersse, Bertrand *et al.*, 2011 ▶). The NLR family encompasses 22 members that are categorized based on their conserved N-terminal domains, all of which belong to the death-domain superfamily. The two largest NLR subfamilies are the caspase activation and recruitment domain (CARD)-containing NLRC family, with four members (NLRC1–NLRC4), and the pyrin domain (PYD)-containing NLRP family, with 14 members (NLRP1–NLRP14) (Kersse, Bertrand *et al.*, 2011 ▶).

NLRP3 is the best-characterized NLRP receptor. Similar to NLRP1 and NLRP6, NLRP3 has been reported to form inflammasome complexes (Martinon *et al.*, 2002 ▶, 2009 ▶). Numerous studies have revealed that these receptors detect pathogen- and danger-associated molecular patterns (PAMPs and DAMPs) and thereby initiate host defence pathways (Schroder & Tschopp, 2010 ▶). NLRPs are held in an auto-inhibited state, as recently described for NLRC4 (Hu *et al.*, 2013 ▶). Upon PAMP or DAMP detection, NLRPs are thought to oligomerize and to recruit the bimodular adapter protein ASC (apoptosis-associated speck-like protein containing a CARD). Through its pyrin domain and CARD, ASC is thought to further recruit the zymogen procaspase-1 to form the inflammasome. The platform-induced oligomerization results in autocatalytic procaspase-1 activation. Active caspase-1 further processes the pro-forms of the pro-inflammatory cytokines IL-1β and IL-18 (Martinon *et al.*, 2002 ▶, 2009 ▶). The important role of NLRPs in innate immunity is underscored by mutations in these receptors that are linked to severe auto-inflammatory diseases such as Muckle–Wells syndrome (Hoffman *et al.*, 2001 ▶).

NLRPs are not restricted to microbial-induced inflammatory signalling pathways. For instance, NLRP2 and NLRP12 have been reported to regulate NF-κB signalling and NLRP4 regulates autophagy (Lich *et al.*, 2007 ▶; Williams *et al.*, 2005 ▶; Fiorentino *et al.*, 2002 ▶; Jounai *et al.*, 2011 ▶). Interestingly, NLRP5, NLRP7 and NLRP14 play important roles in reproduction and development (Tong *et al.*, 2000 ▶; Westerveld *et al.*, 2006 ▶; Murdoch *et al.*, 2006 ▶; Zhang *et al.*, 2008 ▶). NLRP7 has attracted particular interest as it is linked to the formation of hydatidiform moles and resulting miscarriage by affected women (Murdoch *et al.*, 2006 ▶).

On the other hand, mutations in NLRP14 have been described to only affect men, consistent with its testis-specific expression. Several NLRP14 mutations have been reported that have been linked to spermatogenic failure (Westerveld *et al.*, 2006 ▶). A diverse spectrum of endocrine and paracrine factors regulates the highly complex process of spermatogenesis. Utilizing a variety of cytokines and growth factors, immune cells strongly impact testicular function by providing the appropriate microenvironment for spermatogenesis (Hedger, 2002 ▶). Interestingly, IL-1β appears to be a growth factor for the immature, testosterone-producing Leydig cells and serves as a prognostic marker of spermatogenic impairment (Hedger, 2002 ▶; Rozwadowska *et al.*, 2007 ▶).

Structurally, NLRPs share a conserved tripartite domain architecture consisting of an N-terminal pyrin domain, a central NACHT domain (with reference to its initial identification in NAIP, CIITA, Het-E and TP1) and a C-terminal leucine-rich repeat (LRR) domain (Proell *et al.*, 2008 ▶). The ligand-sensing LRR domain switches the receptor from the inactive state to the active state. In the apo state, *i.e.* in the absence of the specific ligand, the LRR is thought to fold back onto the central NACHT domain, which would explain how LRRs can inhibit NACHT-mediated receptor oligomerization (Hu *et al.*, 2013 ▶). Conversely, upon complex formation with a specific ligand (‘ligand sensing’), the LRR is thought to undergo another conformational rearrangement which should lead to NACHT-mediated NLRP oligomerization and activation. This platform is thought to provide the avidity that allows the N-terminal pyrin domain (PYD) to recruit the downstream signalling partner (Martinon *et al.*, 2002 ▶).

Pyrin domains belong to the death-domain superfamily, which includes the CARDs, the death-effector domains (DEDs) and the death domains (DDs) in addition to the PYDs. The death-domain superfamily provides major interaction modules necessary in innate immunity, apoptosis and the necrosis signalling pathway (Kersse, Verspurten *et al.*, 2011 ▶). Given their significance, it is not surprising that viruses have evolved death-domain-containing proteins to interfere with host defence upon virus infection (Hu *et al.*, 1997 ▶; Bertin *et al.*, 1997 ▶; Johnston *et al.*, 2005 ▶). So far, death-domain interactions have only been observed within members of the same death-domain subfamily, referred to as ‘homotypic interactions’ (Reed *et al.*, 2004 ▶). Consequently, receptors containing a CARD domain, such as Apaf-1, can directly recruit and activate CARD-containing effector molecules, *e.g.* caspase-9 (Qin *et al.*, 1999 ▶). By contrast, the homotypic interaction paradigm implies that PYD-containing receptors, *i.e.* NLRPs, require an adaptor molecule that bridges the interaction with caspases. To date, the only known adaptor protein is ASC, which consists of an N-terminal PYD for homotypic interaction with the NLRP receptor and a C-terminal CARD domain to recruit caspase-1 in a homotypic manner.

High-resolution structural information on PYDs is available for the regulatory protein POP1 (pyrin-only protein 1; PDB entry 2hm2), the adaptor protein ASC (PDB entry 1ucp), the DNA-sensing receptor Aim2 (PDB entry 3vd8) and the NLR receptors NLRP1 (PDB entry 1pn5), NLRP3 (PDB entry 3qf2), NLRP4 (PDB entry 4ewi), NLRP7 (PDB entry 2km6), NLRP10 (PDB entry 2m5v) and NLRP12 (PDB entry 2l6a) (Bae & Park, 2011 ▶; Eibl *et al.*, 2012 ▶; Hiller *et al.*, 2003 ▶; Jin *et al.*, 2013 ▶; Liepinsh *et al.*, 2003 ▶; Natarajan *et al.*, 2006 ▶; Pinheiro *et al.*, 2010 ▶, 2011 ▶; Su *et al.*, 2013 ▶). All structures adopt the typical death-domain fold, which is conserved in the entire death-domain superfamily. The death-domain fold consists of six antiparallel α-helices packed around a highly conserved hydrophobic core with Greek-key topology (Steward *et al.*, 2009 ▶). While all death domains share the six-helix bundle architecture, each subfamily reveals distinct structural features. In the case of PYDs, α-helix 3 is characteristically shortened or is even replaced by a long unstructured loop (Hiller *et al.*, 2003 ▶; Pinheiro *et al.*, 2010 ▶, 2011 ▶). Although the structural data based on PYDs are increasing, structural information for a PYD–PYD complex is still lacking.

However, structural information is available for DD–DD, DED–DED and CARD–CARD complexes. Interestingly, all complexes reveal an asymmetric interaction with 1:1 stoichiometry. The complexes can be grouped into three types. The type I interaction as seen in a CARD–CARD complex involves α-helices 1 and 4 of one CARD with α-helices 2 and 3 of the other CARD (Ferrao & Wu, 2012 ▶). The CARD–CARD complex between Apaf-1 and procaspase-9 shows this type I interaction and serves as a model for PYD–PYD complexes, which have so far resisted structural characterization (Eibl *et al.*, 2012 ▶; Jin *et al.*, 2013 ▶; Park, 2012 ▶; Pinheiro *et al.*, 2010 ▶, 2011 ▶).

To decipher the structural principles that might govern the PYD interaction, we set out to determine the crystal structure of NLRP14 PYD. We found an unexpected conformational rearrangement that leads to a symmetric PYD dimerization mode. We discuss the relevance of this unexpected finding for NLRP signalling and for ASC-mediated caspase-1 activation.

## 2. Materials and methods   

### 2.1. Protein expression and purification   

The coding region of NLRP14 PYD (residues Met1–Gln100) was PCR-amplified from human NLRP14 cDNA (GenBank accession No. NM_176822.3) and cloned into the pET-28a stop vector (Novagen), attaching a thrombin-cleavable N-terminal His_6_ tag. Mutations (D86V and L84R) were introduced by Round-the-Horn site-directed mutagenesis. The sequence-verified wild-type and mutated NLRP14 PYD plasmids (Eurofins MWG Operon, Ebersberg, Germany) were transformed into *Escherichia coli* BL21 Star (DE3) cells (Invitrogen). The cells were grown in 600 ml Luria Broth medium at 37°C to an OD_600_ of 0.8, whereupon protein expression was induced with 420 µ*M* isopropyl β-d-1-thio­galactopyranoside (IPTG). After 5 h at 25°C, the cells were harvested by centrifugation and resuspended in lysis buffer (50 m*M* KH_2_PO_4_ pH 7.8, 300 m*M* NaCl, 5 m*M* imidazole, 10% glycerol, 0.1 mg ml^−1^ lysozyme) and stored at −20°C. The His_6_-tagged NLRP14 PYD-containing lysate was clarified by centrifugation and loaded twice onto 800 µl pre-equilibrated Ni^2+^–NTA resin (Qiagen) for immobilized metal-affinity chromatography. After successive washing steps with wash buffer I (20 m*M* Tris pH 8.0, 1 *M* NaCl, 10 m*M* imidazole) followed by wash buffer II (20 m*M* Tris pH 8.0, 1 *M* NaCl, 20 m*M* imidazole), the protein was eluted by thrombin digestion (Sigma–Aldrich). Untagged NLRP14 PYD was further purified by size-exclusion chromatography (SEC) using a Superdex 75 10/300 gel-filtration column (GE Healthcare) in gel-filtration buffer (20 m*M* Tris pH 8.0, 150 m*M* NaCl, 5 m*M* TCEP). The monomer:dimer ratio was quantified by using the appropriate tool from the *UNICORN* software package (GE Healthcare). Fractions containing monomeric protein were pooled and concentrated by centrifugal evaporation for use in subsequent crystallization experiments. Selenomethionine (SeMet)-labelled NLRP14 PYD was expressed in SeMet-substituted minimal medium (Molecular Dimensions) and purified under identical conditions.

### 2.2. Crystallization and data collection   

SeMet-labelled NLRP14 PYD and native NLRP14 PYD mutants (D86V and L84R) were crystallized at 20°C in sitting drops using vapour diffusion. SeMet-labelled crystals were obtained by mixing equal volumes of protein solution (1.0 µl purified NLRP14 PYD at a concentration of 8.5 mg ml^−1^ in gel-filtration buffer) and precipitant solution (1.0 µl 0.1 *M* HEPES pH 7.5, 2.6 *M* ammonium sulfate, 2% PEG 400). NLRP14 PYD D86V was crystallized with a precipitant solution consisting of 0.2 *M* cesium chloride, 2.2 *M* ammonium sulfate and NLRP14 PYD L84R was crystallized with 0.2 *M* ammonium acetate, 2.2 *M* ammonium sulfate at protein concentrations of 5.7 and 8.5 mg ml^−1^, respectively. Crystals formed after 7 d and were prepared for cryo-crystallography by a quick bath in either LV CryoOil (MiTeGen) or 3.4 *M* sodium malonate pH 7.0. Flash-cooled crystals were stored in liquid nitrogen. Diffraction data were collected on beamline BL14.1 at the BESSY II electron-storage ring, Helmholtz Zentrum Berlin für Materialien und Energie and on the microfocus beamline ID23-2 at ESRF Grenoble (Gabadinho *et al.*, 2010 ▶). Data were processed using *iMosflm* v.7.0.6 (Battye *et al.*, 2011 ▶).

### 2.3. Phasing and refinement   

The NLRP14 PYD structure was solved using the 4W-MAD protocol of *Auto-Rickshaw* (Panjikar *et al.*, 2005 ▶). The input diffraction data were prepared and converted for use in *Auto-Rickshaw* using programs from the *CCP*4 suite (Collaborative Computational Project, Number 4, 1994 ▶; Winn *et al.*, 2011 ▶). *F*_A_ values were calculated using *SHELXC* (Sheldrick, 2008 ▶, 2010 ▶). Based on an initial analysis of the data, the maximum resolution for substructure determination and initial phase calculation was restricted to 3.2 Å. 13 out of 16 Se sites were found using *SHELXD* (Schneider & Sheldrick, 2002 ▶). The correct hand for the substructure was determined using *ABS* (Hao, 2004 ▶) and *SHELXE* (Sheldrick, 2010 ▶). The occupancy of all substructure atoms was refined and the initial phases were calculated using *MLPHARE* (Collaborative Computational Project, Number 4, 1994 ▶). The twofold noncrystallographic symmetry (NCS) operator was found using *RESOLVE* (Adams *et al.*, 2010 ▶). Density modification, phase extension and NCS averaging were performed using *DM* (Cowtan, 1994 ▶). A partial α-helical model was produced using *HELICAP* (Nam *et al.*, 2004 ▶). NLRP4 PYD (PDB entry 4ewi; Eibl *et al.*, 2012 ▶) was used as a template for further model building. The partial model contained 385 residues out of the total of 424 residues. Iterative cycles of model building and refinement using *Coot* (Emsley & Cowtan, 2004 ▶) and *PHENIX* (Adams *et al.*, 2010 ▶) led to a final model with an *R*_cryst_ and *R*_free_ of 0.205 and 0.261, respectively, at 2.4 Å resolution (*R*_free_ was calculated using 5% of the reflections, which were randomly omitted from the refinement).

The D86V and L84R mutant structures of NLRP14 PYD were both solved by molecular replacement using *Phaser* from the *CCP*4 suite (McCoy *et al.*, 2007 ▶; Collaborative Computational Project, Number 4, 1994 ▶; Winn *et al.*, 2011 ▶). Wild-type NLRP14 PYD from Ser6 to Pro67 corresponding to α-helices 1–4 served as a model. Thus, calculation of an OMIT map rules out that the interesting α-helix 5–6 region is biased by the atomic model. Finally, iterative cycles of model building and refinement using *Coot* (Emsley & Cowtan, 2004 ▶) and *PHENIX* (Adams *et al.*, 2010 ▶) led to a model of NLRP14 PYD D86V with an *R*_cryst_ and *R*_free_ of 0.216 and 0.268, respectively, at 3.0 Å resolution. The final model of NLRP14 PYD L84R at 2.0 Å resolution was refined to an *R*_cryst_ and *R*_free_ of 0.182 and 0.222, respectively (*R*_free_ was calculated using 5% of the reflections which were randomly omitted from the refinement). Table 1 ▶ summarizes the data-collection, model and refinement statistics of wild-type and mutant NLRP14 PYD. The final model of NLRP14 PYD includes four molecules (chain *A*, Ser7–Ile95; chain *B*, Ser8–Gln100; chain *C*, Ser7–Asn96; chain *D*, Ser6–Ala99).

### 2.4. Protein Data Bank accession code   

The coordinates and structure factors were deposited in the Protein Data Bank (PDB). NLRP14 PYD was deposited with accession code 4n1j, NLRP14 PYD D86V with accession code 4n1k and NLRP14 PYD L84R with accession code 4n1l.

### 2.5. Bioinformatics analysis   

The sequence alignment of NLRP14 PYD with the PYDs of NLRP1–NLRP13 was generated with *MultAlin* (Corpet, 1988 ▶). *PDBsum* was used to created the secondary-structure elements (Laskowski, 2009 ▶). The dimeric interface of NLRP14 PYD and the contact area of helix 6 were analyzed by *PISA* (Krissinel & Henrick, 2007 ▶). Structure visualization and analysis were performed in *PyMOL* (Schrödinger). Electrostatic surface potentials were calculated using the *APBS* plugin in *PyMOL* (Baker *et al.*, 2001 ▶).

### 2.6. Protein characterization using size-exclusion chromatography (SEC) and circular dichroism (CD)   

The oligomerization states of wild-type NLRP14 PYD and its mutants were investigated by SEC on an analytical Superdex 75 10/300 gel-filtration column (GE Healthcare). The SEC column was calibrated with a gel-filtration calibration kit (GE Healthcare) in gel-filtration buffer according to the manufacturer’s protocol. 700 µl of each protein at a concentration of 1 mg ml^−1^ were injected onto the pre-equilibrated column. An excess of 5 m*M* TCEP in the gel-filtration buffer was used to eliminate higher order oligomers arising from disulfide bonding between the only cysteine (Cys88) in NLRP14 PYD.

Secondary-structure elements and the thermal denaturation of NLRP14 PYD and its mutants were determined by CD spectroscopy. Spectra were recorded at 20°C and a protein concentration of 0.1 mg ml^−1^ in 20 m*M* sodium phosphate buffer pH 8.0, 100 m*M* NaCl. The CD spectra were collected using a 0.1 cm path-length cuvette within a JASCO J-815 spectropolarimeter (Jasco, Tokyo, Japan). The thermal denaturation of the proteins was monitored at 222 nm with a temperature gradient of 1°C min^−1^ from 20 to 95°C. The melting temperature (*T*_m_) was calculated from the inflection point of the resulting sigmoid curve. All baseline-corrected spectra are presented as mean residue molar ellipticity [Θ]_MRW_ at a given wavelength or temperature.

### 2.7. Yeast two-hybrid analysis   

The yeast two-hybrid experiments were performed with the Matchmaker GAL4 Two-Hybrid System 3 (Clontech) as described previously. Briefly, NLRP14 PYD (residues Met1–Gln100) or NLRP14 PYD+Linker (residues Met1–Thr190) were inserted into pGADT7 and ASC PYD (residues Met1–Gly94) was inserted into the pGBKT7 fusion vector encoding a reporter GAL-4 DNA-binding domain (BD) and an activation domain (AD), respectively. The *Saccharomyces cerevisiae* reporter strain Y2HGold (Clontech) was co-transformed with pGBKT7-ASC PYD (prey protein) and pGADT7-NLRP14 PYD (bait protein) or the longer construct pGADT7-NLRP14 PYD+Linker (bait protein) using a small-scale lithium acetate/single-stranded carrier DNA/polyethylene glycol (LiAc/ss-DNA/PEG) transformation protocol (Gietz & Woods, 2002 ▶). In a control experiment, the reverse transformation was monitored (pGADT7-ASC PYD and pGBKT7-NLRP14 PYD or pGBKT7-NLRP14 PYD+Linker; Supplementary Fig. S4^1^). Transformed yeast cells were resuspended in sterile water and spotted onto SD/−Leu/−Trp dropout medium to assess the transformation efficiency. Picked clones were diluted as indicated in Fig. 5(*c*) and spotted onto SD/−Leu/−Trp and SD/−Ade/−His/−Leu/−Trp selection medium to test for potential interactions. The plates were incubated at 30°C for 4–5 d. The co-transformation of pGBKT7-ASC PYD and pGADT7-Aim2 PYD (residues Met1–Thr96) served as a positive interaction control (Wagner *et al.*, 2009 ▶). To rule out false-positive interactions, all fusion constructs were tested for their ability to activate transcription of the GAL-4 reporter.

## 3. Results   

### 3.1. NLRP14 PYD adopts an open death-domain fold enabling symmetric dimerization   

**Figure 1:**
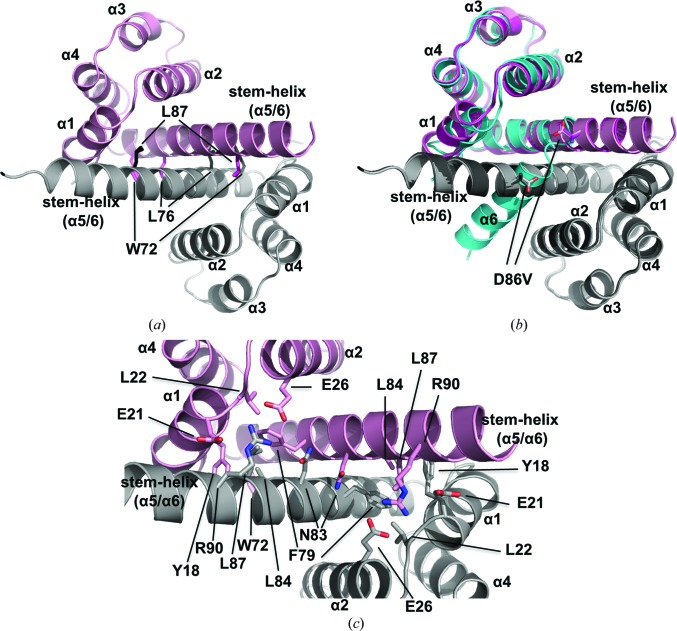
Symmetric dimerization of NLRP14 PYD mediated by an extended α5/6 helix. (*a*) The crystal structure of the wild-type NRLP14 PYD is viewed along the molecular twofold axis (chain *A* in pink; chain *B* in light grey); the important hydrophobic side chains Trp72, Leu76 and Leu87 are shown in stick representation, highlighting their contribution to symmetric α5/6_*A*_–α5/6_*B*_ binding. (*b*) The crystal structure of the physiologically relevant D86V mutant (chain *A* in magenta; chain *B* in dark grey) is superimposed onto the virtually identical structure of wild-type NLRP14 PYD (chain *A* in pink; chain *B* in light grey). Additionally, a model of the canonical six-helix bundle conformation is superimposed (cyan), identifying the conformationally adaptive role of Trp72, Leu76 and Leu87, which can either engage in symmetric dimerization (extended α5/6 stem-helix conformation) or in stabilization of the hydrophobic core (canonical closed conformation). (*c*) Enlarged view of the dimer interface, showing that a variety of hydrophobic, polar and charged interactions stabilize the dimer interface. Dimer contacts are not limited to α5/6_*A*_–α5/6_*B*_ interactions, as exemplified by the monodentate and bidentate interactions of Arg90 (α5/6_*B*_) with Glu21 (α1_*A*_) and Glu26 (α2_*A*_), respectively.

To investigate the functional role of the human NLRP14 PYD, we set out to crystallize the wild-type protein. We succeeded in obtaining diffracting crystals under several distinct conditions. Surprisingly, all three different crystallization conditions for which diffraction data could be preliminarily analysed revealed a dimeric arrangement of NLRP14 PYD (Fig. 1 ▶ *a*). The asymmetric unit is built up as a tetrameric arrangement resulting from a dimerization of dimers (Supplementary Fig. S1). Each of the four crystallographically independent NLRP14 PYD monomer structures adopts a canonical pyrin-domain fold with its N-terminal helices α1–α5. Characteristic of pyrin domains, helix α3 is exposed and only contains two helical turns (Fig. 1 ▶ *a*). By contrast, the typical C-terminal α6 helix is not present as a separate secondary-structure element. Instead, the two C-terminal helices α5 and α6 combine to form an extended stem-helix, which we refer to as stem-helix α5/6. This reordering was found in each of the four crystallographically independent NLRP14 PYDs. It is this extended α5/6 stem-helix that mediates the dimer interface in the NLRP14 PYD crystal structure. The dimer encompasses an extensive interface area of 890 Å^2^.

The α-helical architecture agrees well with known pyrin-domain organizations. Specifically, helix α1 contained Phe10–Glu21, α2 Lys24–Glu39, α3 Leu44–Lys52 and α4 Arg55–Tyr65. The α5/6 stem-helix extended from Lys70 to Asn96. Comparison with other pyrin domains revealed that NLRP14 PYD and NLRP3 PYD share the longest helical conformation for α3.

Remarkably, the pyrin-domain dimer is formed by a symmetric arrangement, contrasting with all previously described types of homotypic death-domain dimerization motifs, which fall into three major classes (Park, 2011 ▶). The α5/6-mediated dimer interaction is stabilized by a hydrophobic motif engaging Trp72, Leu76 and Leu87 from both molecules (Figs. 1 ▶ *a* and 1 ▶ *c*). Importantly, these three residues participate in the formation of the canonical hydrophobic core of pyrin domains. This highly conserved hydrophobic core serves to stabilize the classical six-helix bundle and additionally involves Leu15, Leu19, Leu22, Leu27, Phe30, Leu34, Leu58, Met62, Ala71, Phe79 and Met82. As such, Trp72, Leu76 and Leu87 serve as a hydrophobic core switching element that can engage either in dimer stabilization, as observed in this crystal structure, or, by analogy to classical pyrin domains, in formation of the six-helix bundle (Fig. 1 ▶ *b*).

### 3.2. The physiological NLRP14 PYD D86V mutant adopts an open conformation similar to the wild-type protein   

These findings prompted us to investigate the potential structural impact of the D86V mutant, which is found in men suffering from spermatogenic failure (Westerveld *et al.*, 2006 ▶). To this end, we crystallized the D86V mutant. The crystal structure of the D86V mutant similarly revealed a dimeric arrangement (chain *A* in magenta and chain *B* in dark grey), virtually indiscernible from that of the wild-type protein (chain *A* in pink and chain *B* in light grey) (Fig. 1 ▶ *b*). Consequently, the clinical D86V mutation of NLRP14 PYD apparently does not affect its three-dimensional structure in comparison to the wild-type protein.

### 3.3. NLR PYDs are classified by a charge bridge that acts as a conformational regulation element   

**Figure 2:**
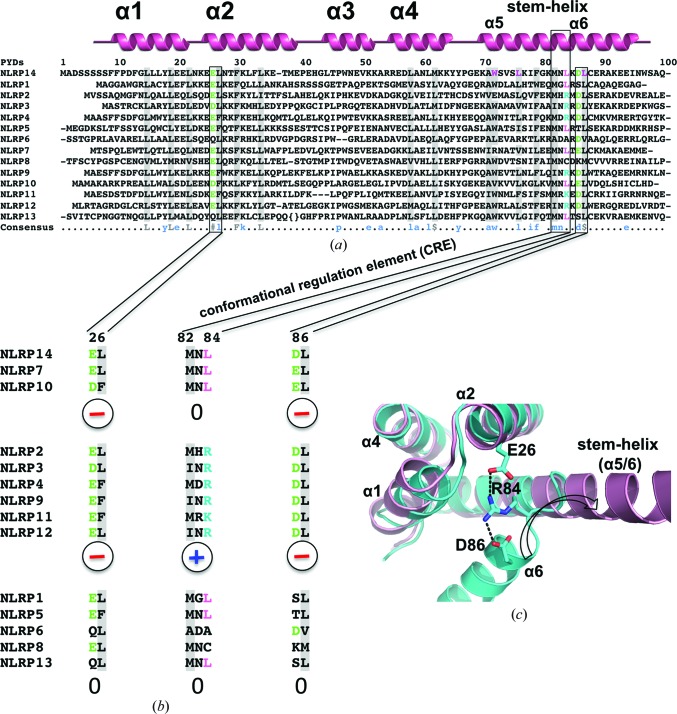
The Glu–Arg–Asp charge relay as an NLRP conformational regulation element. (*a*) Sequence alignment of all 14 NLR PYDs. The secondary-structure elements α1–α6 are indicated. The conserved hydrophobic core residues are highlighted in grey. Additionally, the solvent-exposed hydrophobic residues Trp72, Leu76 and Leu87 are coloured magenta. Brackets indicate the NLRP13-specific insertion of residues Leu41–Gln49. (*b*) Close-up of the consensus of Glu26, Asp86 and the alternative conformational regulation element (CRE) M_82_NL_84_ or M_82_NR_84_. Both Glu26 and Asp86 are necessary for the charge bridge with the MNR motif. (*c*) The crystal structure of NLRP14 PYD L84R (cyan) superimposed onto that of wild-type NLRP14 PYD (pink). The L84R mutant adopts a closed conformation; the intact charge bridge is highlighted.

We next performed a sequential and structural comparison of different NLR pyrin domains to delineate the structural basis for the open *versus* closed conformation. The comparison revealed that a charge bridge, formed by Glu26–Arg84–Asp86, stabilizes the closed pyrin-domain conformation (Figs. 2 ▶ *a* and 2 ▶ *b*). NLRP14 PYD contains Leu at the central position 84, thereby breaking the charge bridge. To test the relevance of the proposed charge bridge to the pyrin-domain conformation, we introduced a L84R mutant to reconstitute the stabilizing charge bridge. The crystal structure of this L84R mutant indeed revealed the closed pyrin-domain conformation, in excellent agreement with our prediction (Fig. 2 ▶ *c*). The introduced positive charge of Arg84 bridges the negative charges of the opposing Glu26 and Asp86. This structure further confirms the proposed adaptive role of the hydrophobic residues Trp72, Leu76 and Leu87 that contribute to the hydrophobic core and thereby stabilize helix α6 within the bundle.

With this crystal structure analysis we have identified a conformational regulation element (CRE) that allows us to predict the tendency of pyrin domains to adopt the closed or open conformation. The latter should correlate with the pyrin-mediated dimerization propensity of the NLRPs. NLRP2, NLRP3, NLRP4, NLRP9, NLRP11 and NLRP12 have an intact charge bridge, resulting in stabilization of the closed six-helical bundle structure (Fig. 2 ▶ *b*).

### 3.4. NLRP14 PYD exists in an equilibrium of predominantly the monomer and dimer in solution   

In the next step, we investigated the dimerization behaviour of NLRP14 PYD in solution. As a side remark, we should point out that Cys88 does not participate in dimer formation in the crystal structure. Additionally, all experiments were performed in the presence of reducing agent (5 m*M* TCEP), preventing spontaneous cysteine oxidation.

**Figure 3:**
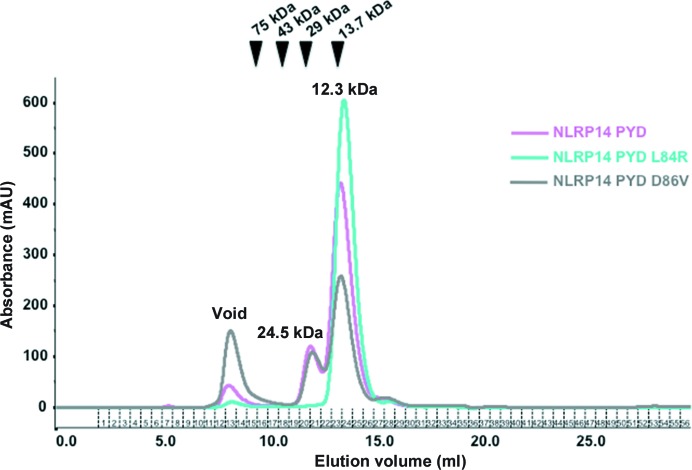
Monomer–dimer distribution of NLRP14 PYD variants as probed by gel-filtration chromatography. Wild-type NLRP14 PYD (magenta) elutes at 12.04 and 13.46 ml, corresponding to dimeric (17%) and monomeric (83%) protein, respectively. NLRP14 PYD D86V (grey) elutes with a qualitatively similar profile (12.13 ml, 24%; 13.48 ml, 76%). By contrast, NLRP14 PYD L84R (cyan) exists exclusively as a monomer in solution (13.63 ml).

Gel-filtration experiments revealed a bimodal distribution for the wild-type pyrin domain, with a dominant monomer (retention volume of 13.46 ml) and a smaller dimer fraction (12.04 ml) (Fig. 3 ▶, pink line). Our previous structure-derived conclusions on the significance of the charge bridge/CRE suggested that the L84R mutant should adopt a stable six-helix bundle conformation. Consistently, we found the L84R mutant to exclusively migrate as a monomer (retention volume of 13.63 ml; Fig. 3 ▶, cyan line). Finally, we also tested the physiological D86V mutant. The charge-bridging system is similarly broken as in the wild-type protein, suggesting that this mutant could undergo the conformational switching necessary for dimerization. Indeed, the D86V mutant showed a very similar dimerization tendency to that observed for the wild-type protein (Fig. 3 ▶, grey line). Remarkably, however, the D86V mutant revealed a higher tendency towards aggregation, as indicated by the pronounced void peak.

We concluded from these experiments that wild-type NLRP14 PYD predominantly exists as a monomer in solution, with a monomer:dimer ratio of approximately 83:17% as judged from chromatogram peak areas (Fig. 3 ▶). Importantly, however, the dimeric fraction is long-lived enough to migrate on the gel-filtration column as a homogenous dimer fraction with a retention volume that corresponds very accurately to a dimer (the duration of the run was ∼40 min). If the dimer were short-lived and with dimer–monomer exchange times within seconds or minutes, a much smaller change in the retention volume would be expected corresponding to the time-averaged size of one molecule rather than the observed bimodal elution distribution. The observed time dependence of the dimer–monomer distribution in wild-type NLRP14 PYD suggests that conformational changes accompany the monomer–dimer transition, consistent with the closed (six-helix bundle) and open (extended α5/6 helix) conformation.

We further asked whether the observed peak ratio of dimer to monomer fractions can be explained by the law of mass action. Therefore, we repeated the gel-filtration runs at a fivefold increased protein concentration (5 mg ml^−1^), as the increase in protein concentration increases the probability of encounter complexes (data not shown). Importantly, however, the distribution of dimer and monomer fractions remained unchanged compared with the gel-filtration run that was carried out at a protein concentration of 1 mg ml^−1^. Apparently, many of the NLRP14 PYD encounter complexes are not productive. Our observations suggest that the limiting factor for NLRP14 PYD dimer formation is the conformational rearrangement to the extended α5/6 stem-helix. The latter is the prerequisite for productive encounter complexes and is hardly influenced by protein concentration.

### 3.5. The NLRP14 L84R mutant reconstitutes the charge bridge, drastically increasing its thermal stability   

**Figure 4:**
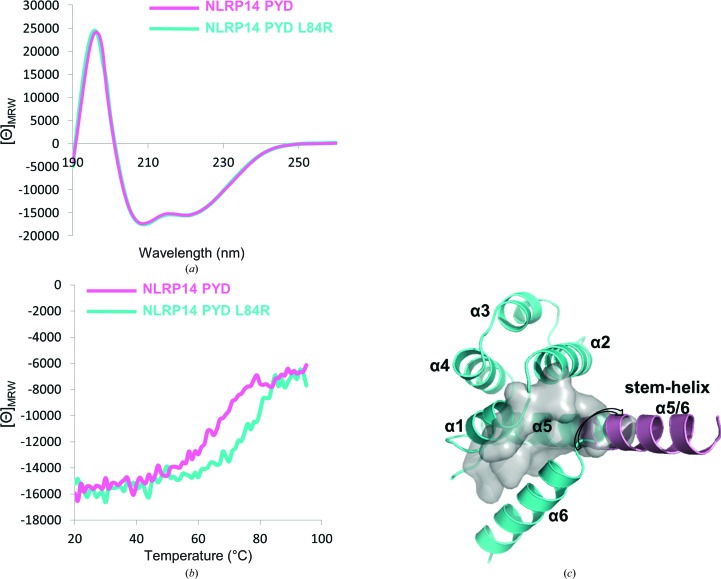
Secondary-structure content and thermal stability of NLRP14 PYD variants. (*a*) Wild-type NLRP14 PYD (pink) and NLRP14 PYD L84R (cyan) reveal a virtually identical CD spectrum, consistent with a nearly identical α-helical content in the open and closed conformation. (*b*) The thermal melting curve of the L84R mutant (cyan) is significantly shifted to higher temperatures compared with the wild-type PYD. This 17° shift corresponds to melting temperatures of 82.6 ± 1.3 and 65.6 ± 0.5°C for the L84R mutant and wild-type NLRP14 PYD, respectively. (*c*) The grey surface area corresponds to approximately 490 Å^2^ of hydrophobic contact area which becomes exposed upon transition from the closed six-helix bundle state (corresponding to the L84R mutant) to a (partially) extended α5/6 helix conformation (wild-type protein).

The gel-filtration experiments imply that the monomeric form of wild-type NLRP14 PYD could exist in different conformational states. We therefore set out to comparatively investigate the conformation of the *bona fide* monomer NLRP14 PYD L84R with the monomeric form of the wild-type protein. To this end, we employed circular-dichroism (CD) spectroscopy to test the folding states of the two variants in solution. CD spectroscopy is particularly well suited to determine the α-helical content of proteins. We hypothesized that the L84R mutant would adopt a six-helix bundle conformation exclusively, whereas the wild-type form might undergo transitions between the extremes of all-open and all-closed states. Importantly, the α-helical content should be virtually identical in the monomeric and dimeric states. According to the crystal structures, the secondary-structure content of the two proteins differs only at the half-helical turn between helix α5 and α6 (Fig. 1 ▶ *b*). Indeed, the CD spectra of the L84R variant and the wild-type protein were virtually identical, indicating an almost identical secondary-structure content in both proteins (Fig. 4 ▶ *a*).

Given the structure analysis (Figs. 1 ▶ *b* and 2 ▶ *c*), we further hypothesized that the thermal stability of the monomeric protein would vary with transition from the open to the closed conformation. Therefore, we carried out thermal melting experiments using CD spectroscopy. The six-helix bundle structure (L84R) exhibited a drastically increased thermal stability (82.6°C) compared with the wild-type protein (65.6°C) (Fig. 4 ▶ *b*). This +17°C difference in melting temperature can only be rationalized when considering the different tertiary helix packing in both proteins. The drastic change in the melting temperature therefore cross-validates the open conformation, as observed in the crystal structure of wild-type PYD14 (Fig. 1 ▶). The L84R variant adopts a canonical death-domain fold with all six helices contributing to the stabilizing hydrophobic core. By contrast, in the wild-type protein the α6 helix has to exhibit some flexibility and will adopt the partially or fully extended α5/6 stem-helix conformation. The α6 helix interface will therefore be mostly uncovered, resulting in partial exposure of the hydrophobic core interface (see Fig. 4 ▶ *c*). In quantitative terms, the hydrophobic core in the canonical six-helix bundle form (L84R) amounts to 2435 Å^2^, whereas the corresponding contact area will be reduced in the wild-type protein by approximately 490 Å^2^ to 1945 Å^2^, *i.e.* an approximate 20% reduction in the stabilizing interaction interface. This is visualized by the grey hydrophobic area that is partially exposed to the solvent in the wild-type protein and in the physiological D86V mutant (*T*_m_ = 57.7°C; Fig. 4 ▶ *c*). We conclude that in the monomeric wild-type NLRP14 PYD the α6 helix is only loosely interacting with helices α1 and α5 of the hydrophobic core. This observation is consistent with a binary conformational space, as sampled by our crystal structures (open and closed; Fig. 1 ▶ *b*). However, the transition between these preferred states will sample conformational intermediates (half-open), although the extent of their occupancy is unclear (Fig. 1 ▶ *b*).

### 3.6. The electrostatic surface potential of dimeric NLRP14 PYD rationalizes the relevance of the D86V mutation   

**Figure 5:**
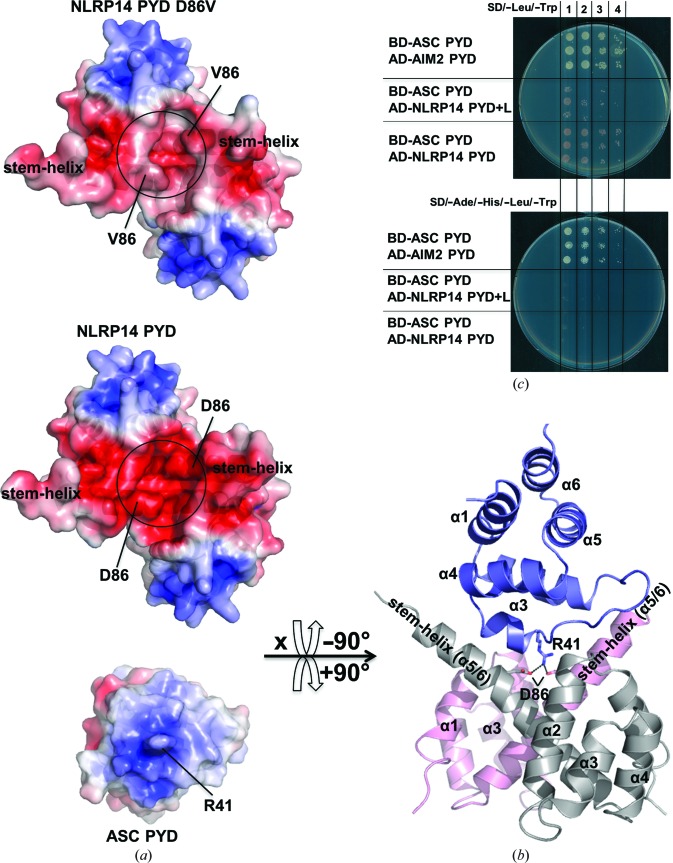
Electrostatic surface potentials of NLRP14 PYD and the physiological D86V mutant reveal significant differences. (*a*) In NLRP14 PYD D86V the extensive negatively charged surface prominent in wild-type NLRP14 PYD is broken by hydrophobic patches. Interestingly, the possible interaction partner ASC presents a complementary charged surface around Arg41 suitable for interaction. (*b*) Proposed 2:1 complex model of NLRP14 PYD (chains *A* and *B* in pink and grey) and ASC PYD (PDB entry 1ucp, blue). Residues mediating the interaction are labelled and shown as sticks. This hypothetical model illustrates how the D86V mutation will affect the binding properties in the dimeric state of NLRP14 PYD. (*c*) NLRP14 PYD does not interact with ASC PYD in a 1:1 complex. NLRP14 PYD and the longer construct NLRP14 PYD+Linker were co-transformed with ASC. The SD/−Leu/−Trp plate confirms that the transformation worked for all combinations. However, the SD/−Ade/−His/−Leu/−Trp plate reveals that neither NLRP14 PYD nor NLRP14 PYD+Linker interacts with ASC PYD. In contrast, Aim2 PYD interacts with ASC in a 1:1 complex and thus demonstrates that the experimental setup worked. Lanes 1, 2, 3 and 4 indicate serial dilutions of 1:1, 1:10, 1:100 and 1:1000.

While the engineered L84R mutant showed remarkable properties such as a drastically improved thermal stability that correlates with a strictly monomeric six-α-helix structure, the naturally occurring D86V mutant behaved very much like the wild-type protein despite its severe physiological implications. This suggested that the mechanistic basis for the physiological role of the D86V mutation should relate to its interaction with other relevant components rather than an intrinsic change within the pyrin-domain structure. Structural comparison of wild-type and D86V pyrin domains showed that the impact of the mutation would be maximized in the dimeric state of the protein because the D86V mutation is positioned very closely to the twofold-symmetry axis of the dimer, thereby doubling the charge-removal effect (Fig. 5 ▶ *a*). This analysis is corroborated by calculation of the electrostatic potential, which reveals a continuous and extensive acidic surface patch for the wild-type dimer protein. By contrast, the negative charge is significantly reduced in this potential interface of the D86V mutant. Although there is no experimental confirmation for NLRP14 PYD, ASC has been proposed to act as an adaptor protein for NLR PYDs (Liepinsh *et al.*, 2003 ▶). The proposed ASC adaptor protein indeed exhibits a pronounced basic surface patch on its pyrin module that would match the dimer-generated acidic contact area (Figs. 5 ▶ *a* and 5 ▶ *b*). Manual docking revealed that the electrostatically steered ASC–(PYD)_2_ complex forms a steric complementary complex (Fig. 5 ▶ *b*). Specifically, this theoretical docking model would predict Arg41 of ASC to be chelated by the two Asp86 residues originating from the NLRP14 PYD dimer. Still, we emphasize that the proposed docking model merely serves to illustrate a possible mechanism for how the D86V mutation can severely affect the interaction of NLRP14 PYD with other binding partner molecules, even if ASC should turn out not to be the physiological binding partner of NLRP14 PYD.

This model not only rationalizes the significance of the D86V mutation by its loss of electrostatic complementarity to ASC (Fig. 5 ▶ *b*) and its tendency for aggregation (Fig. 3 ▶), but it also predicts the necessity of dimeric NLRP14 PYD for ASC binding. We performed yeast two-hybrid screening to investigate whether monomeric PYD could bind ASC (Fig. 5 ▶ *c*, Supplementary Fig. S4). Whereas the positive control clearly revealed the ASC–AIM2 interaction, NLRP14 PYD did not interact with ASC. When assuming that the yeast-expressed NLRP14 PYD is monomeric, the latter finding would be consistent with the proposed stoichiometry of the ASC–(PYD)_2_ complex.

## 4. Discussion   

The surprising finding of a dimeric NLRP14 PYD arrangement prompted us to investigate possible influences by the crystal lattice, which is known to sometimes affect the protein oligomerization state. Additionally, one needs to consider that crystallization usually involves a high protein concentration, which fosters dimerization owing to mass action. In order to minimize effects of the crystallization process (‘crystal artefacts’), we screened for, and identified, different chemical compositions of the crystallization buffer at near-physiological conditions (neutral pH) under which we could grow NLRP14 PYD crystals. All crystal forms showed a dimeric arrangement of the wild-type and D86V pyrin domains. We should note that despite a large variation of chemical space, all wild-type and D86V crystals belonged to the hexagonal space group *P*6_3_ with four molecules per asymmetric unit, resembling a dimer of dimers (Supplementary Fig. S1). By contrast, the L84R mutant crystallized in space group *P*2_1_2_1_2 with one (monomeric) molecule per asymmetric unit.

This markedly different behaviour of wild-type PYD and the engineered L84R variant is reflected in the tendency for dimerization in the wild type and the clinical mutant (D86V) in solution. Nonetheless, the dimer constitutes only a minor fraction as shown by gel-filtration chromatography. Together with the thermal stability experiments, we conclude that the wild-type NLRP14 PYD will be mostly monomeric, with its C-terminal α6 helix contributing to the hydrophobic core only transiently.

Although rare, a five-helical core constitutes a stable structural building block, as demonstrated by the five-helix death-domain conformations in the Fas–FADD complex, prolegumain and NLRC1 (Srimathi *et al.*, 2008 ▶; Dall & Brandstetter, 2013 ▶; Scott *et al.*, 2009 ▶). This structural precedence underlines the significance of the surprising extended α5/6 stem-helix. Clearly, crystal structures are sampling discrete states in the dynamically accessible conformational space continuum. Therefore, the interpretation of structure–function relationships often calls for additional biochemical experiments to support the structural conclusions (Wang *et al.*, 2010 ▶). In this work, we could back up the crystal structure findings with complementary experiments demonstrating that the predicted dimerization state exists in solution and critically depends on Leu84 (Figs. 3 ▶ and 4 ▶ *b*), as predicted by the crystal structure analysis (Fig. 2 ▶).

The very high protein concentration during the crystallization process is one likely driver for the observed dimerization of wild-type NLRP14 PYD. This consideration gives an important hint towards the possible physiological role of the observed pyrin dimerization. The NLRP14 protein is organized as a mosaic protein whereby the N-terminal pyrin domain is followed by a NACHT domain and a C-terminal LRR region. NACHT domains are known to undergo oligomerization and will thus bring two pyrin domains into close proximity (Martinon *et al.*, 2002 ▶). The crystal structure analysis additionally suggests that the proximity of the NACHT domain will also induce the extended α5/6 helix rearrangement and thus stimulate the dimerization of the N-terminal pyrin domains. This dimerization generates unique binding epitopes that are not present in monomeric NLRP14 PYD (Fig. 5 ▶ *a*).

Given the similarity of the critical charge-relay system in NLRP14, NLRP7 and NLRP10 (Fig. 2 ▶ *b*), it is tempting to speculate whether these three NLRPs share an identical dimerization-induction mechanism. In analogy to our results with NLRP14 PYD (Fig. 3 ▶), we predict that isolated NLRP7 PYD and NLRP10 PYD will be mostly monomeric, consistent with NMR studies on these pyrin domains (Pinheiro *et al.*, 2010 ▶; Su *et al.*, 2013 ▶). As a side remark, the fact that the NMR data showed the NLRP7 PYD and NLRP10 PYD to be exclusively in the closed state may reflect a bias that is intrinsic to the NMR method: in an ensemble where different α6 helix conformations are populated, including the canonical closed conformation and more open conformations resembling the extended α5/6 stem-helix, the closed conformation will be preferentially detected by NMR because only in this case will short distances (<5–6 Å) to neighbouring atoms be present which can result in NOE signals. As a consequence, the PYDs of NLRP7, NLRP10 and NLRP14 may functionally act as dimers upon NACHT-induced oligomerization. The complex formation with a binding partner, as illustrated by the ASC–(PYD)_2_ docking model (Fig. 5 ▶ *b*), may additionally contribute to (PYD)_2_ stabilization. On the other hand, NLRP2, NLRP3, NLRP4, NLRP9, NLRP11 and NLRP12 have an intact charge-relay element (Fig. 2 ▶ *b*) and thus should maintain the monomeric closed six-helix bundle conformation even upon NACHT oligomerization.

It will therefore be important to take the stoichiometry of pyrin complexes explicitly into account in the design of future experiments. In particular for NLRP7, NLRP10 and NLRP14, dimeric or oligomeric stoichiometries of pyrin-domain complexes must be explored.

By adopting symmetric pyrin-domain homodimerization, nature may follow a quantitative and a qualitative aim. Firstly, the signal transduction by NLRP7, NLRP10 and NLRP14 occurs in a dampened way, down-regulating the incoming signal by a factor of 2. As an example, a hexameric NLRP14 complex would be able to recruit only three caspases, with a corresponding reduction in interleukin 1β production. Secondly, the symmetric pyrin dimer observed here generates a qualitatively new binding interface that cannot be resembled by two monomers. Consequently, we propose that the (PYD)_2_ dimer breaks with the death-domain paradigm whereby only homotypic death-domain interactions can occur (Kersse, Verspurten *et al.*, 2011 ▶). The interactome of the proposed (PYD)_2_ dimers in NLRP7, NLRP10 and NLRP14 might expand outside of the pyrin-domain family and even outside the death-domain superfamily.

## Supplementary Material

Supporting Information.. DOI: 10.1107/S1399004714010311/mv5102sup1.pdf

PDB reference: NLRP14 PYD, 4n1j

PDB reference: D86V mutant, 4n1k

PDB reference: L84R mutant, 4n1l

## Figures and Tables

**Table 1:** Data-collection and refinement statistics Values in parentheses are for the highest resolution bin.

	NLRP14 PYD					
	λ1 MADSe	λ2 MADSe	λ3 MADSe	λ4 MADSe	NLRP14 PYD D86V	NLRP14 PYD L84R
Data collection						
Space group	*P*6_3_				*P*6_3_	*P*2_1_2_1_2
Unit-cell parameters (Å, °)	*a* = *b* = 89.61, *c* = 107.90, α = β = 90.0, γ = 120.0				*a* = *b* = 89.21, *c* = 106.60, α = β = 90.0, γ = 120.0	*a* = 51.35, *b* = 62.55, *c* = 29.11, α = β = γ = 90.0
Synchrotron, beamline	BESSY II, PX14.1				BESSY II, PX14.1	ESRF, ID23-2
Wavelength (Å)	0.97973	0.97626	1.00801	0.97985	0.91841	0.87260
Resolution (Å)	53.93–2.60 (2.79–2.60)	44.86–2.54 (2.68–2.54)	44.90–2.63 (2.77–2.63)	41.42–2.55 (2.69–2.55)	44.60–3.00 (3.16–3.00)	39.69–1.99 (2.09–1.99)
Mosaicity (°)	0.37	0.60	0.80	0.80	0.58	1.36
No. of reflections	114721 (14803)	120880 (16105)	108426 (13333)	118067 (14559)	73663 (10800)	44825 (6289)
Unique reflections	14400 (1868)	15450 (2155)	13998 (1797)	15255 (1979)	9722 (1418)	6909 (988)
Completeness (%)	94.7 (84.4)	94.9 (91.6)	94.5 (83.8)	95.0 (85.2)	100.0 (100)	100.0 (100.0)
Multiplicity	8.0 (7.9)	7.8 (7.5)	7.7 (7.4)	7.7 (7.4)	7.6 (7.6)	6.5 (6.4)
Mean *I*/σ(*I*)	8.9 (1.8)	9.4 (1.3)	9.3 (1.2)	9.8 (1.6)	9.7 (1.8)	9.5 (3.2)
*R*_meas_	0.13 (1.26)	0.13 (1.96)	0.13 (1.84)	0.12 (1.35)	0.20 (1.53)	0.14 (0.67)
CC_1/2_ (high-resolution shell)	0.63				0.49	0.72
Model and refinement statistics						
Resolution range (Å)	44.81–2.60				44.61–3.00	39.69–1.99
No. of reflections (total)	14374				9697	6879
No. of reflections (working)	13652				9230	6551
Completeness (%)	94.70				99.92	99.96
*R*_work_	0.209				0.216	0.184
*R*_free_ †	0.254				0.268	0.222
Ramachandran plot (%)						
Favoured	97.3				96.6	100.0
Allowed	100.0				100.0	100.0
Stereochemical parameters						
R.m.s.d., bond lengths (Å)	0.004				0.002	0.007
R.m.s.d., bond angles (°)	0.802				0.811	1.029
Wilson *B* value (Å^2^)	60.73				67.61	22.14
ESU based on *R*_free_ value‡	0.40				0.68	0.17
PDB code	4n1j				4n1k	4n1l

† *R*_free_ is defined as for *R*_work_ but is calculated for 5.0% of the total reflections chosen at random and omitted from refinement.

‡ ESU is the estimated overall coordination error (Collaborative Computational Project, Number 4, 1994 ▶; Tickle *et al.*, 1998 ▶)

## Footnotes

1 Supporting information has been deposited in the IUCr electronic archive (Reference: MV5102).
