# Supplementary material for: Structures of the NLRP14 pyrin domain reveal a conformational switch mechanism regulating its molecular interactions
Source: Acta Crystallogr D Biol Crystallogr. 2014 Jun 29;70(Pt 7):2007–18. doi: 10.1107/S1399004714010311 (PMC4089490; doi:10.1107/S1399004714010311)
Supplement: Supplementary file 1 [file d-70-02007-sup1.pdf]

Acta Crystallographica Section D

Volume 70 (2014)

Supporting information for article:

Structures of NLRP14 pyrin domain reveal a conformational switch mechanism, regulating its molecular interactions

Clarissa Eibl, Manuel Hessenberger, Julia Wenger and Hans Brandstetter

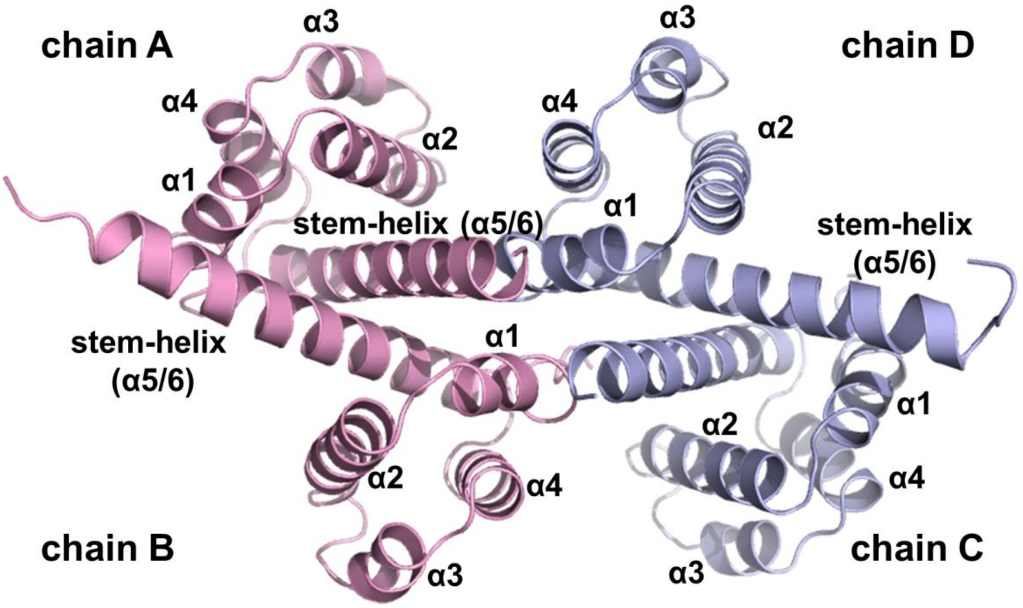

Figure S1 Tetrameric arrangement of NLRP14 PYD in the asymmetric unit. The asymmetric unit consists of four molecules, wherein chain A and B (pink) and chain C and D form an almost identical Dimer (rmsd of 0.45 Å). Whereas the intradimeric interface buries a surface of 890 $Å^2$ the interdimeric interface (between chain A and D and chain D and C) buries 550 $Å^2$ in total. A diagonal interaction between chain A and C is mainly formed by residue Ile95 of both chains.

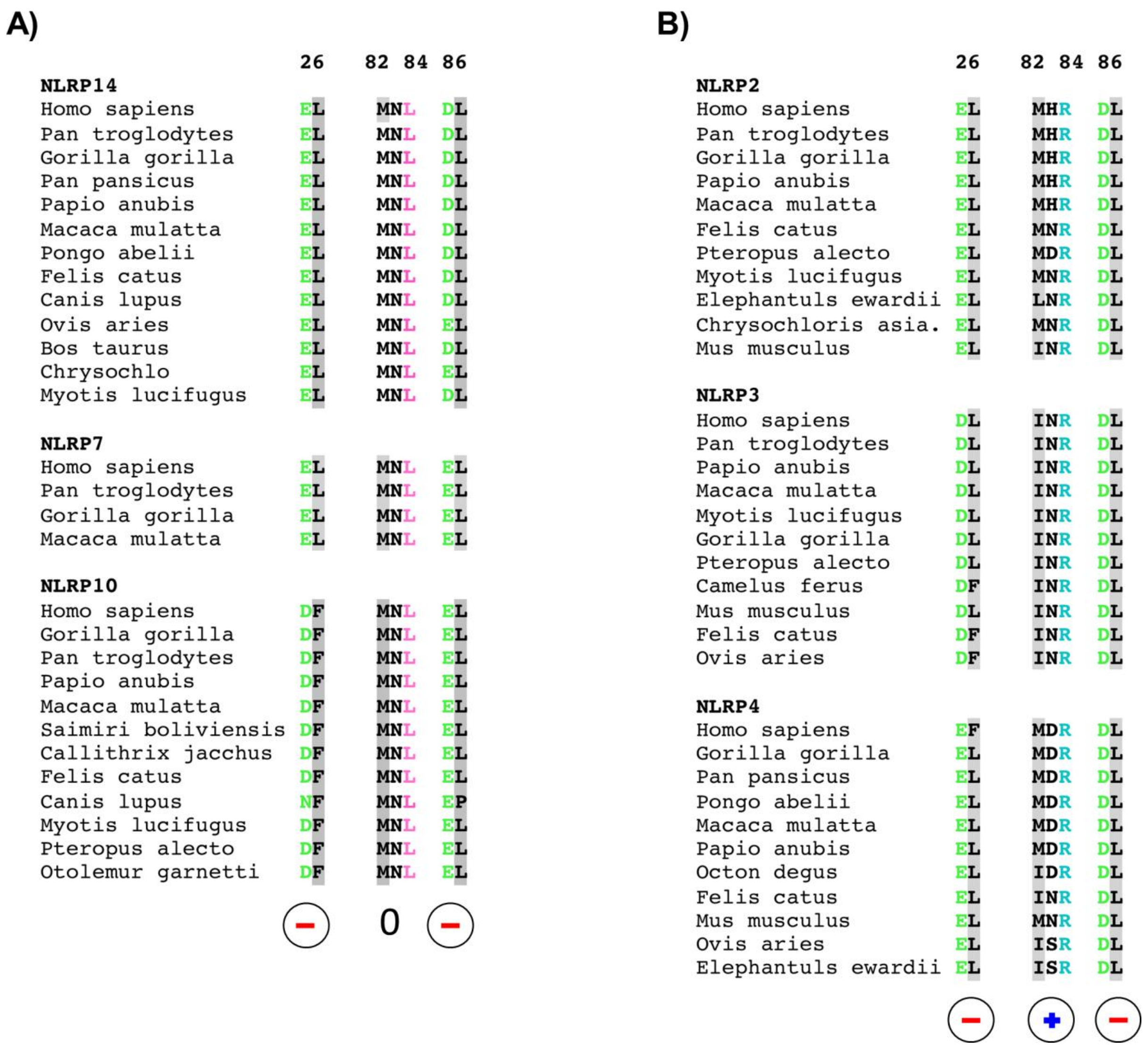

Figure S2 Conservation of the CRE motif in NLR pyrin domains. A) NLRP14, NLRP7 and NLRP10 present a conserved broken charge bridge. B) In contrast the charge bridge is strictly intact for NLRP2, NLRP3 and NLRP4 pyrin domain.

A)

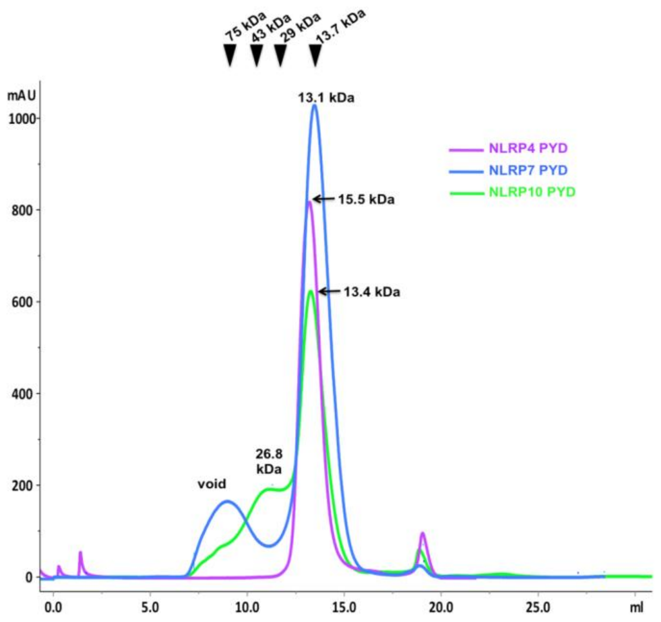

B)

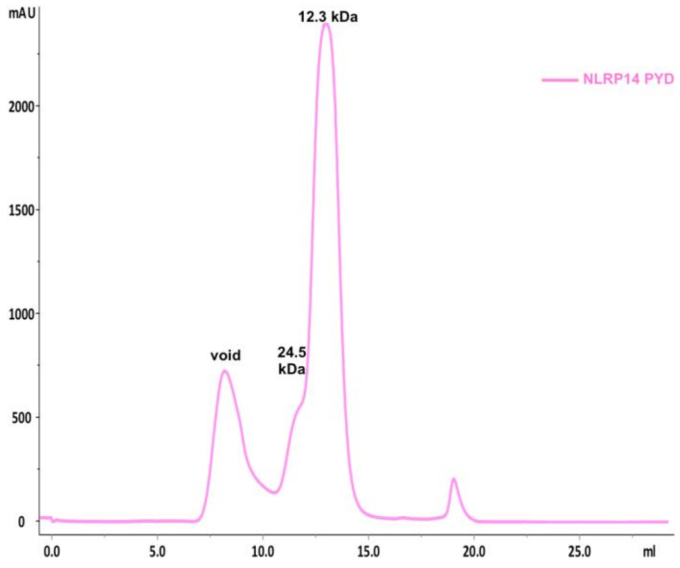

Figure S3 (A) Monomer-dimer distribution of the pyrin domains of NLRP4, NLRP7 and NLRP10 As expected NLRP4 PYD (purple), with an intact charge bridge, exists as a monomer in solution exclusively, whereas NLRP10 PYD (green) reveals a similar monomer-dimer distribution (76% to 24%) as NLRP14 PYD wt. NLRP7 PYD (blue) elutes within one peak, consistent with a monomeric conformation. The absorption differs for the three proteins despite identical concentrations due to the distinct extinction coefficients (NLRP4 PYD, 22460; NLRP7 PYD, 22000; NLRP10 PYD, 12100). (B) Monomer-dimer distribution of NLRP14 PYD at higher concentration. The monomer to dimer ratio is largely independent from the protein concentration (cf. Figure 3), as would be expected from the law of mass action. Apparently, the limiting factor for dimerization is the number of productive molecular encounters which is related to the open α5/6 helix.

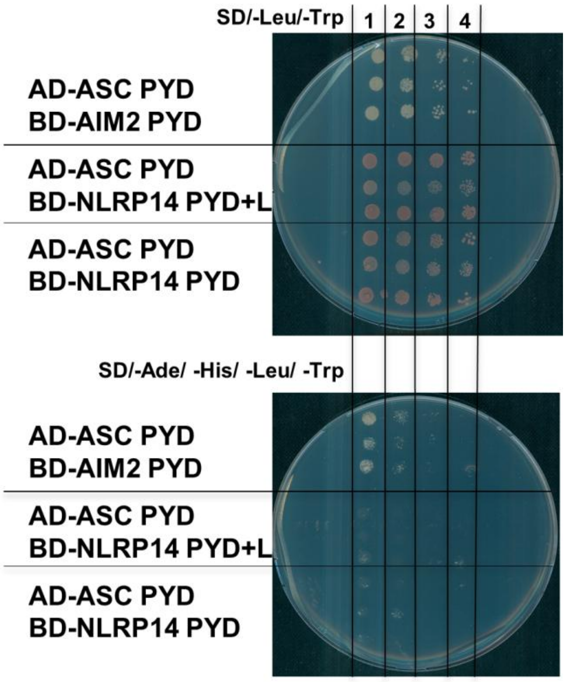

Figure S4 NLRP14 PYD does not interact with ASC PYD in a 1:1 complex. NLRP14 PYD and the longer construct NLRP14 PYD+Linker were co-transformed with ASC. The SD/–Leu/-Trp plate confirms that the transformation worked for all combinations. However, the SD/-Ade/-His/-Leu/-Trp plate reveals that neither NLRP14 PYD nor NLRP14 PYD+Linker interacts with ASC PYD. In contrast, Aim2 PYD interacts with ASC in a 1:1 complex and thus demonstrates that the experimental set up worked. Supplementary Figure S4 corresponds to Figure 5C in the reverse setting, i.e. ASC and NLRP14 PYD(+Linker) swapped. Lane 1, 2, 3 and 4 indicate the serial dilution of 1:1, 1:10, 1:100 and 1:1000.
